# A refined method for analysis of 4,4′-dicofol and 4,4′-dichlorobenzophenone

**DOI:** 10.1007/s11356-017-8956-y

**Published:** 2017-04-06

**Authors:** Ge Yin, Ioannis Athanassiadis, Åke Bergman, Yihui Zhou, Yanling Qiu, Lillemor Asplund

**Affiliations:** 1 Department of Environmental Science and Analytical Chemistry, Stockholm University, SE-10691 Stockholm, Sweden; 2 State Key Laboratory of Pollution Control and Resource Reuse, College of Environmental Science and Engineering, Tongji University, Shanghai, 200092 China; 3 Swedish Toxicology Sciences Research Center, Forskargatan 20, SE-15136 Södertälje, Sweden; 4 Key Laboratory of Yangtze River Water Environment (Ministry of Education), College of Environmental Science and Engineering, Tongji University, Shanghai, 200092 China

**Keywords:** Kelthane, On-column injection, α-Cl-DDT, Cod, Analysis

## Abstract

The acaricide, dicofol, is a well-known pesticide and partly a substitute for dichlorodiphenyltrichloroethane (DDT). Only few reports on environmental occurrence and concentrations have been reported calling for improvements. Hence, an analytical method was further developed for dicofol and dichlorobenzophenone (DCBP) to enable assessments of their environmental occurrence. Concentrated sulfuric acid was used to remove lipids and to separate dicofol from DCBP. On-column injection was used as an alternative to splitless injection to protect dicofol from thermal decomposition. By the method presented herein, it is possible to quantify dicofol and DCBP in the same samples. Arctic cod (*Gadus morhua*) were spiked at two dose levels and the recoveries were determined. The mean recovery for dicofol was 65% at the low dose (1 ng) and 77% at the high dose (10 ng). The mean recovery for DCBP was 99% at the low dose (9.2 ng) and 146% at the high dose (46 ng). The method may be further improved by use of another lipid removal method, e.g., gel permeation chromatography. The method implies a step forward in dicofol environmental assessments.

## Introduction

A number of studies have shown that 1,1-dichloro-2,2-bis(4-chlorophenyl) ethane (4,4′-DDE), the major metabolite of 1,1′-bis(4-chlorophenyl)-2,2,2-trichloroethane (4,4′-DDT), is present at high levels in different environmental media in China (Grung et al. 2015; Yin et al. 2015; Zhang et al. 2013; Zhou et al. 2016a) even though technical DDT was prohibited for agricultural use in 1983 (Yu et al. 2005). Qiu et al. (2005) stated that the use of dicofol in China has become an important source of DDT after the prohibition. This can be explained by the manufacturing process of 4,4′-dicofol being synthesized from DDT. China was the largest consumer of dicofol globally, from 2000 to 2012, with a cumulative usage of 19,500 MT, occupying 69% of the global total amount (Li et al. 2015).

Dicofol (trade name, Kelthane) has been widely used as an organochlorine acaricide that is applied to protect citrus and cotton cultivations from mites (Thiel et al. 2011; Vonier et al. 1996). Dicofol is produced from technical DDT through a pathway including chlorination to form the intermediate 1,1-bis(4-chlorophenyl)-1,2,2,2-tetrachloroethane (α-Cl-DDT), and followed by hydrolysis thereof to form dicofol (Qiu et al. 2005). It is estimated that 93% of 4,4′-DDT is converted to 4,4′-dicofol while only 37% of 2,4′-DDT is converted to 2,4′-dicofol probably due to steric hindrance for the formation of 2,4′- α-Cl-DDT (Qiu et al. 2004). The lower reactivity of 2,4-DDT results in an increased ratio of 4,4′-dicofol/2,4′-dicofol to around 10 (Qiu et al. 2004). Hence, dicofol analyzed in the present study refers to 4,4′-dicofol.

Due to its structural similarity to DDT, dicofol is considered to be of similar concern as DDT and its metabolites DDE and dichlorodiphenyldichloroethane (DDD). These concerns relate to persistence, bioaccumulation, environmental long-range transport, and adverse effect in wildlife and humans (UNEP 2015a). In 2011, the Japanese government rejected the import of Chinese-produced eel due to high levels of dicofol residues, reflecting a severe contamination of this acaricide in China (Wang et al. 2011a).

Dicofol has been listed as a persistent toxic compound in a series of multilateral agreements, e.g., Convention on Long-range Transboundary Air Pollution Protocol on persistent organic pollutants (POPs), and banned in many developed countries (Li et al. 2015). Dicofol has been proposed (2013) as a candidate for POPs in the Stockholm Convention (UNEP 2015a). However, the inclusion of dicofol among the legacy POPs is controversial. For example, there is a lack of evidence of dicofol’s environmental stability. Dicofol is not stable under alkaline condition when it decomposes to dichlorobenzophenone (DCBP) (UNEP 2015a). Dicofol has been demonstrated to easily undergo transformation to DCBP when classical gas chromatography (GC) injection techniques, such as high-temperature split/splitless injectors, are used (Fujii et al. 2011).

The environmental research on dicofol and exposure to dicofol is limited. It is mainly restricted by analytical difficulties. Fujii et al. (2011) used GC coupled to mass spectrometry (MS) in splitless mode to compare dicofol and its related pesticides in breast milk from China, Korea, and Japan assuming dicofol decomposed to DCBP completely. However, according to our experience, dicofol cannot be expected to be quantitatively transformed to DCBP. In addition, it is known that other acaricides, e.g., chlorobenzilate and chloropropylate, may form DCBP (Knowles and Ahmad 1971). Even DDT itself is metabolically transformed to DCBP (Heberer and Dünnbier 1999). Accordingly, dicofol is not the only source of DCBP.

Wiemeyer et al. (2001) used GC with an electron capture detector (ECD) for analysis of dicofol residues in eggs and carcasses of captive American kestrels (*Falco sparverius*). The authors injected dicofol in a temperature-programmed mode at several concentration levels of the analyte and measured the corresponding DCBP amount being formed. They then corrected for dicofol residues in the samples. However, the decomposition ratios are suspected to vary among single injections. Consequently, the use of classical GC injection techniques is problematic for the analysis of dicofol.

Other pretreatment and instrumental techniques, e.g., reversed-phase high-performance liquid chromatography (Han et al. 2011), spectrophotometry (Pandey et al. 2015), and molecularly imprinted solid-phase extraction (Wang et al. 2011a), have been applied according to the scientific literature.

In the present study, a simple solution for improved analysis of dicofol and DCBP is proposed by using an on-column injection technique. The sample was injected directly onto the GC column at a temperature below the boiling point of the solvent. The advantage with this technique is that little or no thermal degradation occurs compared to what happens when splitless injections are done. The applicability of the method is evaluated by the recovery of dicofol and DCBP in spiked cod samples.

## Material and methods

### Solvents and chemicals

The solvents were all of the highest quality available on the market. All glassware were heated at 300 °C overnight before being used. Aluminum oxide (90 active basic, 0.063–0.200 mm) was supplied from Merck and heated to 130 °C overnight prior to use. Cod (*Gadus morhua*) muscle from the North Atlantic was bought from ICA Supermarket, Sweden, for the recovery study.

**Fig. 1:**
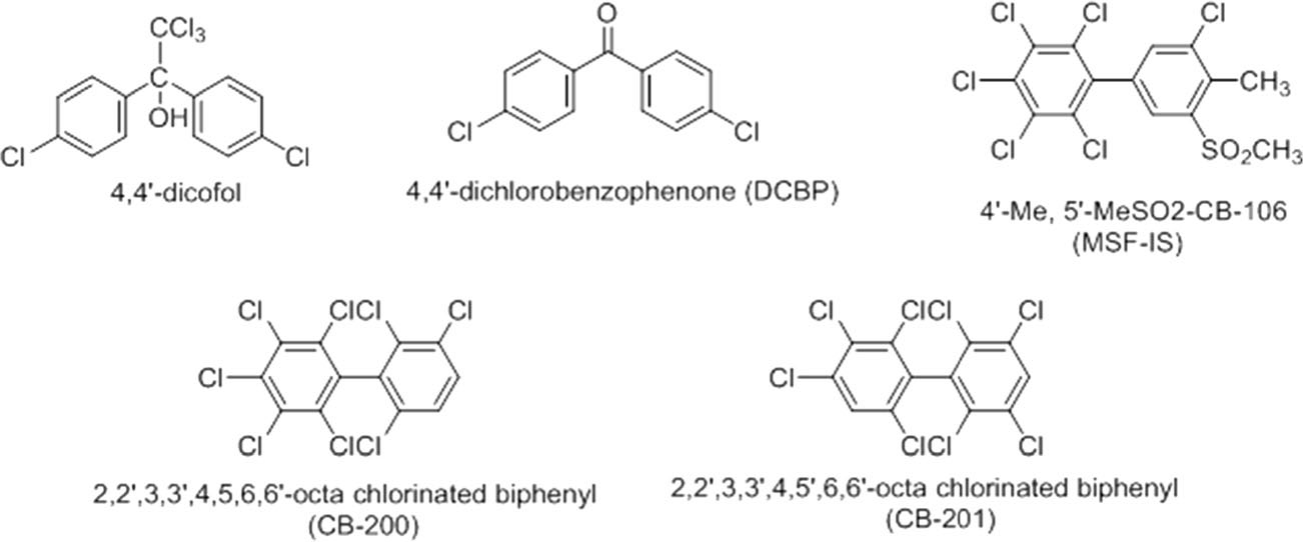
Chemical structure of contaminants involved in the study

The chemical structure of analytes involved in the present study is shown in Fig. 1. Dicofol (99%) and DCBP (98.9%) were purchased from Dr. Ehrenstorfer GmbH, Germany. 2,3,3′,4,4′,5,5′-Heptachlorobiphenyl (CB-189), 2,2′,3,3′,4,5,6,6′-octachlorobiphenyl (CB-200), and 2,2′,3,3′,4,5′,6,6′-octachlorobiphenyl (CB-201) were purchased from AccuStandard (New Haven, USA). 4′-Me-5′-MeSO_2_-CB106 (MSF-IS) was synthesized in-house.

### Recovery study

The recovery study included both dicofol and DCBP. Dicofol was spiked to cod samples at two levels (1 and 10 ng). DCBP, with two levels (9.2 and 46 ng), was spiked to another batch of cod samples. The lower level of each compound is approximately around ten times the limit of quantification whereas the higher level is still in the linear range of ECD. For each compound and concentration level, five replicates were analyzed. In addition to those 20 samples spiked with standards, three cod samples were left unspiked, as control samples. CB-200 and MSF-IS, which have been commonly used in our research group as internal standard (Hovander et al. 2006; Zhou et al. 2016b), were added to the samples as surrogate standards for dicofol and DCBP, respectively. CB-201 was added as volumetric standard prior to analysis for recovery calculation.

### Extraction and clean-up

**Fig. 2:**
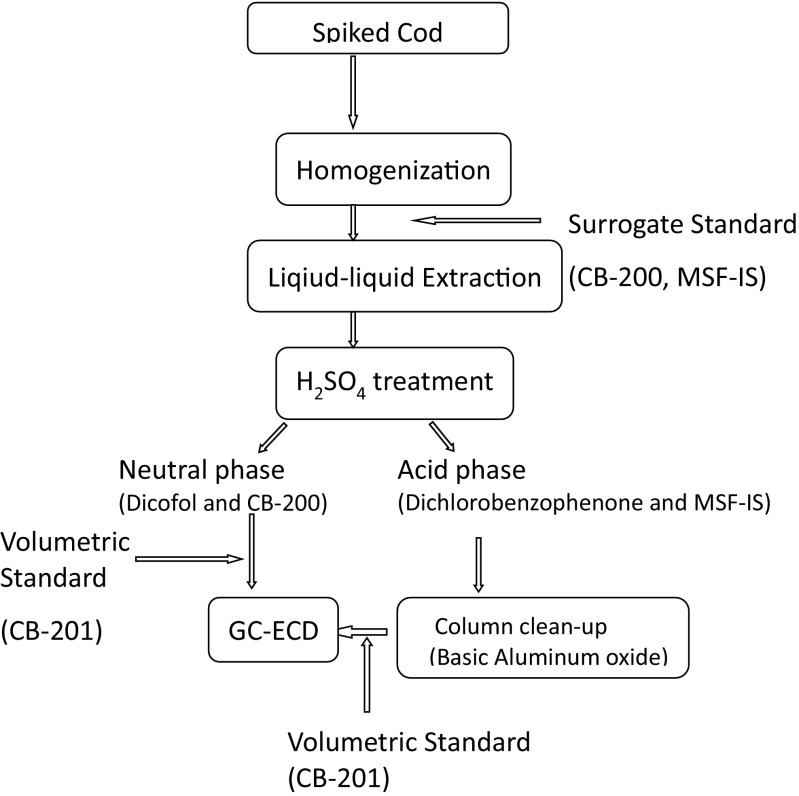
Scheme for analysis of the analytes used in the recovery study

A scheme for the analytical procedure applied is presented in Fig. 2. Cod samples were homogenized with a steel blender as a pool. Extraction was performed according to the Jensen refined extraction method of lean fish with a minor modification that iso-hexane replaced with *n*-hexane (Jensen et al. 2009). One portion (approximately 8 g) of homogenized cod muscle was weighed and then transferred to a centrifuge tube containing isopropanol (IPR, 25 mL) and iso-hexane/diethyl ether (DEE) (3:1, *v*/*v*, 20 mL). Target analyte and surrogate standard were spiked to the sample (Table 1). After homogenization and centrifugation, the extracts were poured into a separatory funnel (150 mL). Re-extraction was performed using IPR (10 mL) and iso-hexane/DEE (40 mL) and the extracts were combined in a separatory funnel. The extracts were then washed with a mixture of sodium chloride (0.9%) in hydrochloric acid (0.2 M hydrochloric acid, 50 mL), inverted 30 times and let stand for phase separation. After 1 h, the water phase was removed to an E-flask. The funnel was washed with the sodium chloride solution (20 mL) again and the separation procedure was then repeated. Finally, the organic phase was collected into a preweighted beaker. The beaker was left in the fume hood at room temperature overnight for evaporation until a stable lipid weight was reached. The lipid was dissolved with iso-hexane (4 mL) and transferred to a 15-mL test tube. Lipid was removed by conc. sulfuric acid (2 mL). The iso-hexane phase was taken out whereas the acid phase was re-extracted with iso-hexane (3 mL) and the combined organic phase was evaporated to 0.2 mL for dicofol analysis.

**Table 1:** Recovery (%) of 4,4′-dicofol (*Difocol*), 4,4′- dichlorobenzophenone (*DCBP*), and their corresponding surrogate standards, 2,2′,3,3′,4,5,6,6′-octachlorobiphenyl (CB-200) and 4′-Me-5′-MeSO_2_-CB106 (MSF-IS), respectively, in cod samples

Dose spiked	Dicofol	CB-200	Dicofol	CB-200	DCBP	MSF-IS	DCBP	MSF-IS
	1 ng	2 ng	10 ng	2 ng	9.2 ng	2.5 ng	46 ng	2.5 ng
Cod 1	77	89	78	97	133	142	151	117
Cod 2	68	76	67	93	62	82	140	96
Cod 3	56	62	82	100	90	138	152	119
Cod 4	62	74	69	89	100	100	154	131
Cod 5	65	84	81	99	108	123	132	92
Mean	65	77	75	96	99	117	146	111
SD	8	10	7	4	26	25	9	17

The test solution was spiked with 1 ng dicofol and 9.2 ng DCBP for the low dose and 10 ng and 46 ng for the high dose, respectively

### Extraction of DCBP

DCBP acts as a Lewis base and partitions into the conc. sulfuric acid in the lipid removal treatment step and needs to be re-extracted from the acid. The acid phase fraction was placed in an ice-bath and diluted with cold distilled water (2 mL) which was subsequently extracted with iso-hexane (3 mL) twice. The combined iso-hexane phase was concentrated to 0.5 mL by a gentle nitrogen flow. The samples were further cleaned up on a basic aluminum oxide column (0.9 g), packed in a Pasteur pipette. The column was preconditioned with *n*-hexane/DEE (9:1, *v*/*v*, 5 mL). The sample was applied to the column and analytes eluted with *n*-hexane/DEE (9:1, *v*/*v*, 12 mL), and thereafter with DEE (10 mL). The solvent volume was reduced by a gentle flow of nitrogen. Prior to instrumental analysis, CB-201 was added as the volumetric standard. The final volume was adjusted to 0.1 mL for GC-ECD analysis.

### Instrumental analysis

A Varian 450-GC equipped with a Varian CP-8400 autosampler and a Varian 1079 programmed temperature vaporizing (PTV) inlet was used. To be able to do an on-column injection, a 2-m non-polar methyl deactivated capillary precolumn (J&W Scientific) with an inner diameter of 0.53 mm, big enough to allow a 26-gauge needle tip to enter the bore, is press-fit connected to an injector liner that is tapered in the middle. The liner is positioned in such a way that the needle tip releases the sample inside the precolumn bore. Automated injections of 1 μL were made in splitless mode, and the inlet temperature of the injector was programmed as follows: initial temperature of 55 °C for 2 min, a 200 °C/min ramp to a final temperature of 280 °C which was maintained throughout the analysis; compressed air was used to cool the injector to the starting temperature. The analytical column (TR-5MS, 30 m × 0.25 mm × 0.1 μm, Thermo Scientific) was connected to the precolumn with a zero dead volume adapter. Helium was used as carrier gas at a constant flow of 1.2 mL/min. The temperature program for the oven started at 55 °C for 2 min, a 15 °C/min ramp up to 300 °C, and maintained there for 8 min. An ECD was used for detection and operated at 325 °C with nitrogen as make-up gas (25 mL/min).

### Quality control

One procedural solvent blank was used in each batch of five samples. Three unspiked cod samples (control samples) were analyzed in parallel with the spiked samples. Neither dicofol nor DCBP was detected in any procedural blank or control sample. The replicate samples were mixed and analyzed in different batches to avoid any systematic bias or contamination.

## Results and discussion

**Fig. 3:**
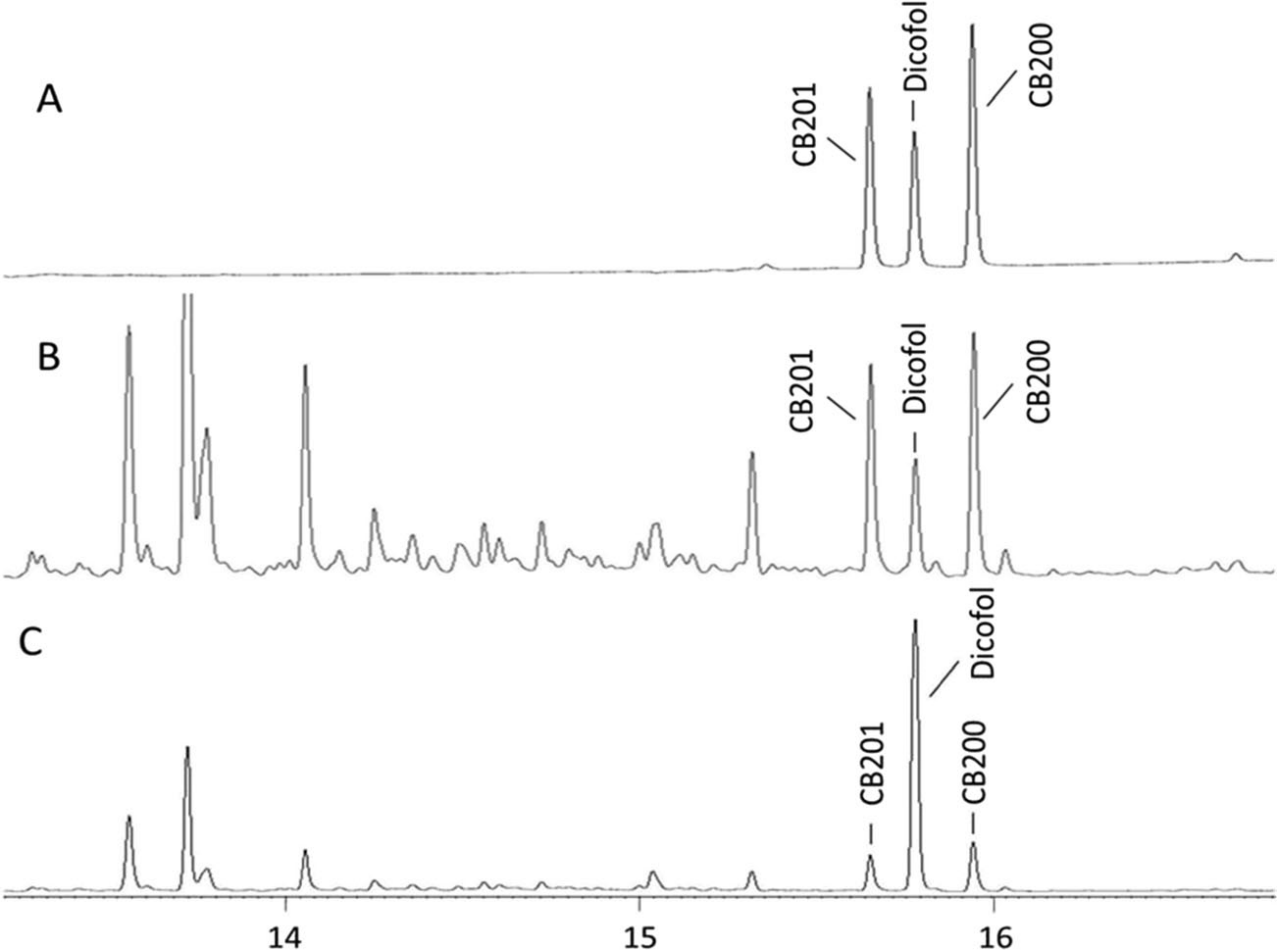
GC-ECD chromatogram of 4,4′-dicofol (*Dicofol*) and 2,2′,3,3′,4,5,6,6′-octachlorobiphenyl (*CB200*) recovery study on cod by on-column injection. **a** *Dicofol* reference. **b** *Dicofol* low dose (1 ng). **c** *Dicofol* high dose (10 ng). *CB201* spiked as volumetric standard

In order to validate the method, a recovery study was conducted in cod. The recovery of dicofol and DCBP is presented in Table 1. The mean recovery of dicofol was 65 ± 8% (56–77%) at low dose (1 ng) and 75 ± 7% (67–82%) at high dose (10 ng), which are regarded as a satisfying result. Figure 3 shows the chromatogram of dicofol by using on-column injection. No DCBP was observed during the instrumental analysis in any spiked sample or control sample. The recovery of CB-200 was 77 ± 10% (62–89%) for low dose and 96 ± 4% (89–100%) for high dose. The good recovery of CB-200 at the two levels indicates that dicofol can be analyzed together with other PCB congeners.

**Fig. 4:**
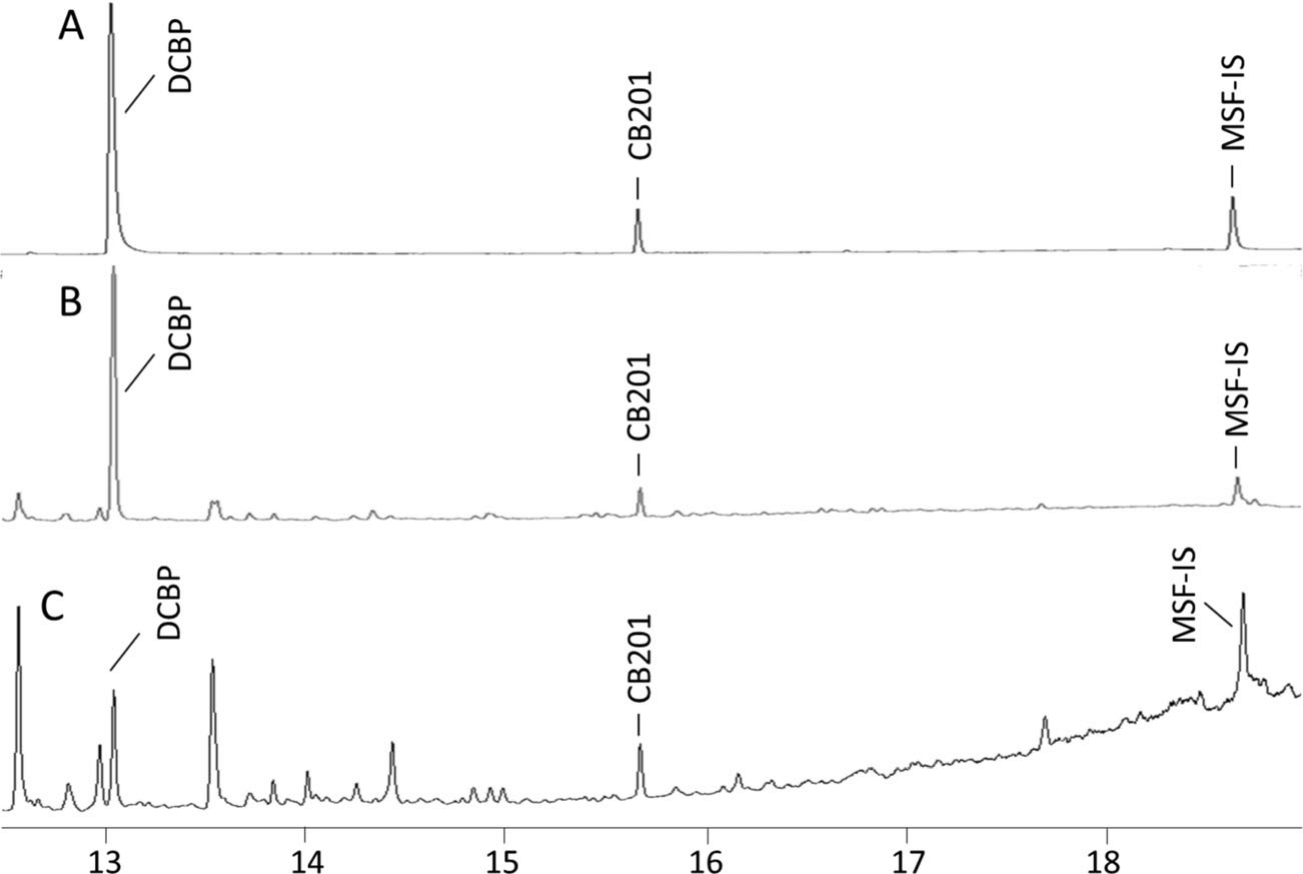
GC-ECD chromatogram of 4,4′-dichlorobenzophenone (*DCBP*) and 4′-Me-5′-MeSO_2_-CB106 (*MSF-IS*) recovery study of cod performed by on-column injection. **a** *DCBP* reference. **b** *DCBP* high dose (46 ng). **c** *DCBP* low dose (9.2 ng). *CB201* spiked as volumetric standard

The recovery of DCBP was 99 ± 26% (62–133%) and 146 ± 9% (132–152%) at low and high doses, respectively (Table 1). The recovery of MSF-IS was 117 ± 25% (82–142%) and 111 ± 17% (92–131%) at low and high doses, respectively. The reason for such difference in the recovery of DCBP may be due to an unstable baseline in the chromatography (Figure 4). The volumetric standard CB-201 elutes in the early part of the slope and MSF-IS elutes at the top of the slope. DCBP elutes in the early part of chromatography; hence, the peak abundance would not be interfered by the slope, but quantification would be influenced when CB-201 was used as an internal standard. The results indicate that MSF-IS is not an optimal surrogate standard for DCBP analysis.

**Fig. 5:**
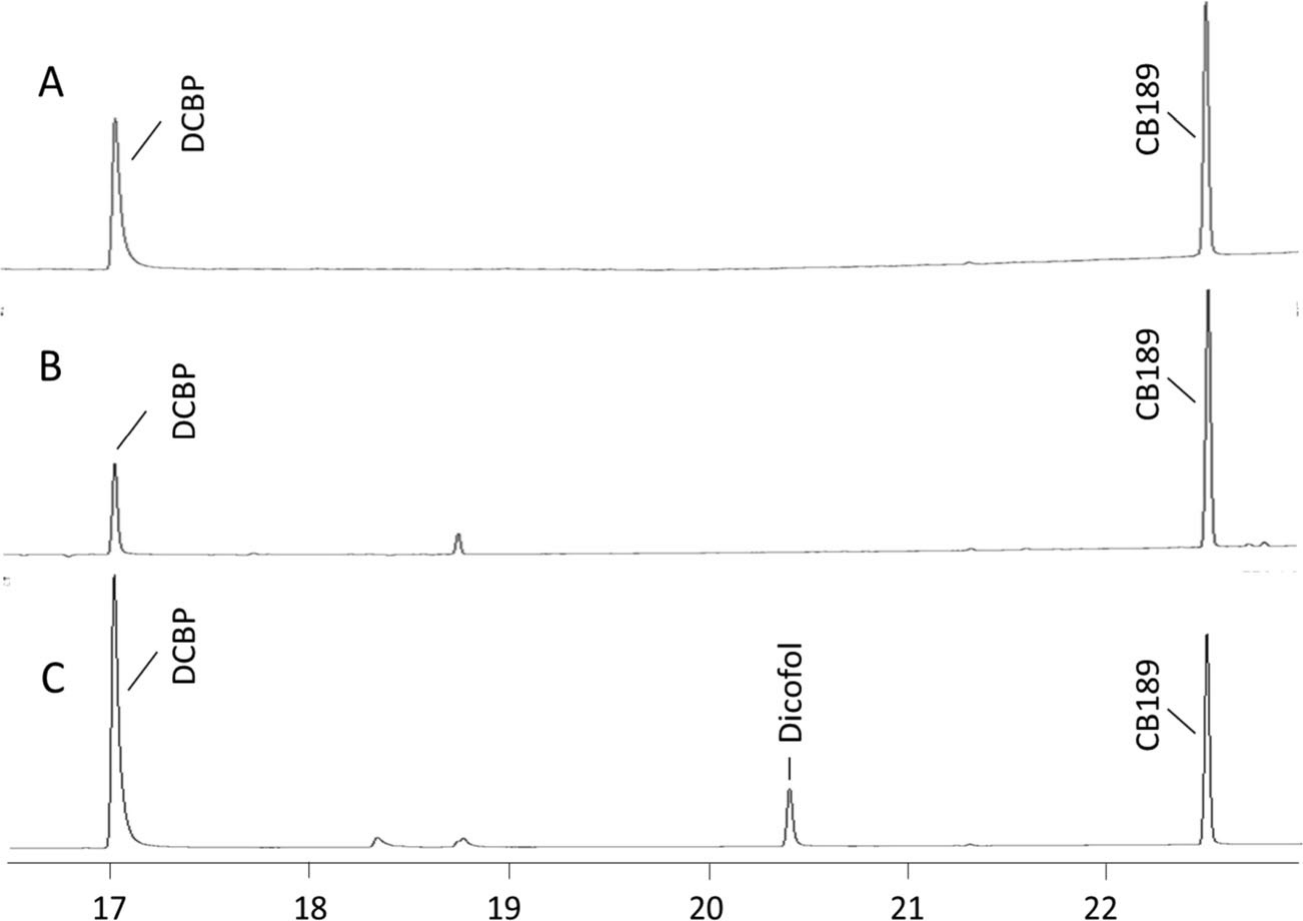
GC-ECD chromatogram of 4,4′-dichlorobenzophenone (*DCBP*) and 4,4′-dicofol (*Dicofol*) injected on a standard split/splitless injector. **a** *DCBP* reference. **b** *Dicofol* transformation to *DCBP* by potassium hydroxide treatment. **c** *Dicofol* reference. *CB189* used as retention time standard

Prior to the recovery test, a number of pretests were performed. Figures 3a and 5c promote a comparison of the two GC chromatograms resulting from dicofol injected in the standard splitless mode (Fig. 5c) and in the on-column mode (Fig. 3a). It is apparent that dicofol is transformed to DCBP when the injections are done in the splitless mode (Fig. 5c). Furthermore, some extra unknown peaks appear in the chromatograms which have yet to be identified. The instrumental parameter and condition affect the occurrence of those peaks, resulting in low reproducibility. Therefore, it is not recommended to use classical split-splitless injector for dicofol analysis. However, if dicofol was injected on an on-column injector, no DCBP was observed in the chromatograms (Fig. 3a). Hence, the solution to the apparent problem is to eliminate sample contact with glass inserts. Testing revealed that dicofol degraded to DCBP on all instances when the sample was introduced to the analytical column via a glass liner, no matter the injection technique used. Those tested were the following: (1) hot splitless injection where the sample is vaporized inside a glass liner at elevated temperatures; (2) temperature-programmed injection where the sample is released inside a glass liner at a start temperature below or at the boiling point of the solvent and then heated rapidly; or (3) direct injection by the use of a tapered glass liner where the sample is deposited inside the walls of the glass insert and forced onto the analytical column by raising the temperature rapidly. Different techniques require also different types of glass liners, and different brands and trademarks were tested, all leading to the same final result. Our conclusion is accordingly to inject the sample directly inside the bore of the analytical column which was achieved by utilizing a precolumn with an inner diameter big enough to accept the size of a syringe.

Since the half-life of dicofol in the environment is shorter compared to legacy POPs, e.g., DDT (UNEP 2015b), it is doubtful that dicofol can be detected in the environmental compartment in its own form. It has been reported that dicofol can undergo photolysis to form DCBP (Chen et al. 1984) and also transform under alkaline conditions (Walsh and Hites 1979). In addition, it has been found that dicofol could be metabolically transformed to DCBP in rats and mice (Brown et al. 1969; Brown and Casida 1987). These findings might explain why dicofol has been rarely detected in environmental studies (c.f. Table 2).

**Table 2:** Summary of dicofol residue in environmental samples reported in the scientific literature

Matrix	Extraction	Clean-up	Instrument	Target ions	Detection limit	Recovery	Residue level	Location	Reference
Apple	Liquid extraction	Column chromatography	GC-ECD		5 ng/g	93–96	n.d.	India	(Singh et al. 2009)
Mother’s milk	Liquid extraction	GPC, silica gel column	GC-MS	139, 250	0.2 ng/g	91	5.8–64 (9.6) ng/g l.w.	China	(Fujii et al. 2011)
Mother’s milk	Liquid extraction	GPC, silica gel column	GC-MS	139, 250	0.2 ng/g	91	0.8–3.0 (1.9) ng/g l.w.	Korea	(Fujii et al. 2011)
Mother’s milk	Liquid extraction	GPC, silica gel column	GC-MS	139, 250	0.2 ng/g	91	<0.1–2.7 (0.32) ng/g l.w.	Japan	(Fujii et al., 2011)
Tea	Microwave-assisted steam distillation	SPE	GC-ECD		0.2 ng/g	81–110	53–990 ng/g	China	(Ji et al. 2007)
Tomato	Liquid extraction	Florisil column	GC-ECD		0.007 μg/g	87	0.002–0.4 μg/g	Morocco	(Salghi et al. 2012)
Human adipose tissue	Liquid extraction	GPC	GC-MS-MS	250, 139			2.91 ng/g fat	China	(Wang et al. 2011b)
Soil	Liquid extraction		GC-ECD		1.85 ng/g	84	11 ng/g	Pakistan	(Syed and Malik 2011)
Soil	Ultrasonic-extraction	Florisil column	GC-MS	250, 139	1.35 ng/g		11 ng/g	China	(Lv et al. 2010)
Egg	Liquid extraction	Acid silica	GC-ECD		6 ng/g	70–98	29–36 ng/g	Pakistan	(Malik et al. 2011)
Egg	Liquid extraction	Acid silica	GC-ECD		6 ng/g	70–98	n.d.—21.6 ng/g	Pakistan	(Malik et al. 2011)

Conc. sulfuric acid is commonly used for lipid removal for POP analysis. However, this step will partition Lewis bases to the conc. sulfuric acid phase, a property first applied in environmental analysis to isolate aryl methyl sulfone compounds, as methylsulfonyl-PCBs (MeSO_2_-PCBs) from other neutral organohalogen compounds (Jensen and Jansson 1976). Also, dibenzophenone compounds are Lewis bases that are partitioning to the conc. sulfuric acid phase in a partitioning with e.g. hexane. Since the sulfuric acid has been commonly discarded, this can explain why DCBP is rarely detected and reported from actual environmental samples.

Alternatives on pretreatment may be considered to further improve the method proposed herein. Cod was used for the recovery study as it has low contaminant levels and low lipid content, the latter to minimize matrix effects. Species with high lipid content require use of larger quantities of conc. sulfuric acid to remove the lipids, which may influence the recoveries. Therefore, other lipid removal methods, e.g., gel permeation chromatography or acetonitrile partitioning, might be used to remove the lipids prior to conc. sulfuric acid treatment. According to the literatures (c.f. Tables 2 and 3), the most common techniques for dicofol analysis are GC-ECD and GC-MS (Fujii et al. 2011; Malik et al. 2011; Wang et al. 2011b; Wiemeyer et al. 2001). There is a possibility to explore conversion of dicofol to DCBP by treatment with sodium hydroxide after conc. sulfuric acid treatment. By this alternative method, dicofol and DCBP are separated by sulfuric acid and then dicofol can be transformed to DCBP by sodium hydroxide treatment before classical GC analysis by split/splitless injector (Fig. 5b). A significant disadvantage with sodium hydroxide treatment compared with on-column injection is that it is limiting the method to become only dicofol-specific. Therefore, the method presented herein is preferable.

**Table 3:** Summary of method for dicofol analysis reported elsewhere

Matrix	Extraction	Clean-up	Instrument	Parent ions	Detection limit	Recovery	Special technique	Spiked amount	Reference
Cod	Liquid extraction	Sulfuric acid basic aluminum column	GC-ECD		0.01 ng/g l.w.	56–81	On-column injection	1 and 10 ng	Present study
Minced samples	Liquid extraction	MISPE	GC-ECD		0.1 ng/g	86–101	Molecularly imprinted solid-phase extraction	1–10 ng/g	(Wang et al. 2011a)
Water, milk, tomato, beans, grapes			UV-visible spectrophotometer			94–99	Spectrophotometric	15–25 μg	(Pandey et al. 2015)
Fish	Liquid extraction	NH_2_-SPE	GC-MS	139 (250, 141)	1 ng/g	79–87		0.02–0.1 ng/g	(Chen et al. 2009)
Orange		Silica gel	GC-ECD		70 ng/g	87–95		0.5–10 μg/g	(Ribeiro et al. 2000)
Tea	Liquid extraction	Dispersive solid phase extraction (d-SPE)	GC-MS UHPLC-MS-MS	250, 252, 251, 254	0.09 μg/g	107	UHPLC-MS-MS		(Zhang et al. 2010)

## Conclusion

In the present study, a method composed of liquid extraction, sulfuric acid clean-up and separation, on-column injection, was developed for dicofol and its major decomposition product DCBP. Dicofol does not break down when on-column injection is used. It should be pointed out that the application of the method may not be limited to analysis of dicofol. On-column injection can be used for compounds that are thermo-labile.

The sulfuric acid treatment can separate chemicals containing a ketone group from neutral compounds in addition to removing lipids. However, improvements and/or alternatives are suggested, e.g., use gel permeation chromatography to remove lipids before sulfuric acid, further clean-up for DCBP phase. The method is a step forward in dicofol environmental assessment and particularly promising to be applied to those environmental samples from dicofol hot spot areas.

## References

[CR1] Brown MA, Casida JE (1987). Metabolism of a dicofol impurity alpha-chloro-DDT, but not dicofol or dechlorodicofol, to DDE in mice and a liver microsomal system. Xenobiotica.

[CR2] Brown JR, Hughes H, Viriyano S (1969). Storage, distribution, and metabolism of 1,1-bis(4-chlorophenyl)-2,2,2-trichloroethanol. Toxicol Appl Pharmacol.

[CR3] Chen ZM, Zabik MJ, Leavitt RA (1984). Comparative-study of thin-film photodegradative rates for 36 pesticides. Industrial & Engineering Chemistry Product Research and Development.

[CR4] Chen SB, Yu XJ, He XY, Xie DH, Fan YM, Peng JF (2009). Simplified pesticide multiresidues analysis in fish by low-temperature cleanup and solid-phase extraction coupled with gas chromatography/mass spectrometry. Food Chem.

[CR5] Fujii Y, Haraguchi K, Harada KH, Hitomi T, Inoue K, Itoh Y (2011). Detection of dicofol and related pesticides in human breast milk from China, Korea and Japan. Chemosphere.

[CR6] Grung M, Lin Y, Zhang H (2015). Pesticide levels and environmental risk in aquatic environments in China—a review. Environ Int.

[CR7] Han SY, Qiao JQ, Zhang YY (2011). Determination of n-octanol/water partition coefficient for DDT-related compounds by RP-HPLC with a novel dual-point retention time correction. Chemosphere.

[CR8] Heberer T, Dünnbier U (1999). DDT metabolite bis(chlorophenyl) acetic acid: the neglected environmental contaminant. Environmental Science & Technology.

[CR9] Hovander L, Linderholm L, Athanasiadou M (2006). Levels of PCBs and their metabolites in the serum of residents of a highly contaminated area in eastern Slovakia. Environmental Science & Technology.

[CR10] Jensen S, Jansson B (1976). Anthropogenic substances in seal from the baltic: methyl sulfone metabolites of PCB and DDE. Ambio.

[CR11] Jensen S, Lindqvist D, Asplund L (2009). Lipid extraction and determination of halogenated phenols and alkylphenols as their pentafluorobenzoyl derivatives in marine organisms. J Agric Food Chem.

[CR12] Ji J, Deng CH, Zhang HQ, Wu YY, Zhang XM (2007). Microwave-assisted steam distillation for the determination of organochlorine pesticides and pyrethroids in Chinese teas. Talanta.

[CR13] Knowles CO, Ahmad S. (1971) Comparative metabolism of chlorobenzilate, chloropropylate, and bromopropylate acaricides by rat hepatic enzymes. Can J Physiol Pharmacol 49:590-& 10.1139/y71-076 5088463

[CR14] Li L, Liu JG, Hu JX (2015). Global inventory, long-range transport and environmental distribution of dicofol. Environmental Science & Technology.

[CR15] Lv JG, Shi RG, Cai YM, Liu Y, Wang ZH, Feng JM (2010). Assessment of 20 organochlorine pesticides (OCPs) pollution in suburban soil in Tianjin, China. Bull Environ Contam Toxicol.

[CR16] Malik RN, Rauf S, Mohammad A, Eqani SAMA, Ahad K (2011). Organochlorine residual concentrations in cattle egret from the Punjab Province, Pakistan. Environ Monit Assess.

[CR17] Pandey GP, Singh AK, Deshmukh L, Asthana A (2015). Determination of dicofol in various environmental samples by spectrophotometric method using chromogenic reagent. Synthesis and Reactivity in Inorganic Metal-Organic and Nano-Metal Chemistry.

[CR18] Qiu XH, Zhu T, Jing L (2004). Organochlorine pesticides in the air around the Taihu Lake, China. Environmental Science & Technology.

[CR19] Qiu XH, Zhu T, Yao B, Hu JX, Hu SW (2005). Contribution of dicofol to the current DDT pollution in China. Environmental Science & Technology.

[CR20] Ribeiro ML, Amador JR, Polese L, Jardim EFG, Minelli EV, de Cordis OCP (2000). Effect of a pilot washing system on dicofol levels in orange matrix. J Agric Food Chem.

[CR21] Salghi R, Luis G, Rubio C, Hormatallah A, Bazzi L, Gutierrez AJ (2012). Pesticide residues in tomatoes from greenhouses in Souss Massa Valley, Morocco. Bull Environ Contam Toxicol.

[CR22] Singh SB, Mukherjee I, Maisnam J, Kumar P, Gopal M, Kulshrestha G (2009). Determination of pesticide residues in integrated pest management and nonintegrated pest management samples of apple (*Malus pumila* Mill.). J Agric Food Chem.

[CR23] Syed JH, Malik RN (2011). Occurrence and source identification of organochlorine pesticides in the surrounding surface soils of the Ittehad Chemical Industries Kalashah Kaku, Pakistan. Environmental Earth Sciences.

[CR24] Thiel A, Guth S, Bohm S, Eisenbrand G (2011). Dicofol degradation to p,p'-dichlorobenzophenone—a potential antiandrogen. Toxicology.

[CR25] UNEP. 2015a. The new POPs under the Stockholm Convention

[CR26] UNEP. 2015b. Evaluation of dicofol against the criteria of Annex D

[CR27] Vonier PM, Crain DA, McLachlan JA, Guillette LJ, Arnold SF (1996). Interaction of environmental chemicals with the estrogen and progesterone receptors from the oviduct of the American alligator. Environ Health Perspect.

[CR28] Walsh PR, Hites RA (1979). Dicofol solubility and hydrolysis in water. Bull Environ Contam Toxicol.

[CR29] Wang H, Yan HY, Qiu MD, Qiao JD, Yang GL (2011). Determination of dicofol in aquatic products using molecularly imprinted solid-phase extraction coupled with GC-ECD detection. Talanta.

[CR30] Wang N, Shi LL, Kong DY, Cai DJ, Cao YZ, Liu YM (2011). Accumulation levels and characteristics of some pesticides in human adipose tissue samples from Southeast China. Chemosphere.

[CR31] Wiemeyer SN, Clark DR, Spann JW, Belisle AA, Bunck CM (2001). Dicofol residues in eggs and carcasses of captive American kestrels. Environ Toxicol Chem.

[CR32] Yin G, Asplund LM, Qiu YL (2015). Chlorinated and brominated organic pollutants in shellfish from the Yellow Sea and East China Sea. Environ Sci Pollut Res.

[CR33] Yu G, Niu JF, Huang J. 2005. Persistent organic pollutants: a new global environmental issue (in Chinese)

[CR34] Zhang XA, Mobley N, Zang JG, Zheng XM, Lu L, Ragin O, et al. 2010. Analysis of agricultural residues on tea using d-SPE sample preparation with GC-NCI-MS and UHPLC-MS/MS. J Agric Food Chem 58:11553−+ 10.1021/jf102476m 20961040

[CR35] Zhang K, Wei YL, Zeng EY (2013). A review of environmental and human exposure to persistent organic pollutants in the Pearl River Delta, South China. Sci Total Environ.

[CR36] Zhou YH, Asplund L, Yin G (2016). Extensive organohalogen contamination in wildlife from a site in the Yangtze River Delta. Sci Total Environ.

[CR37] Zhou YH, Yin G, Asplund L (2016). A novel pollution pattern: highly chlorinated biphenyls retained in black-crowned night heron (*Nycticorax nycticorax*) and whiskered tern (*Chlidonias hybrida*) from the Yangtze River Delta. Chemosphere.

